# Functional characterisation of naturally occurring mutations in human melanopsin

**DOI:** 10.1007/s00018-018-2813-0

**Published:** 2018-04-26

**Authors:** Jessica Rodgers, Stuart N. Peirson, Steven Hughes, Mark W. Hankins

**Affiliations:** Nuffield Laboratory of Ophthalmology, Sleep and Circadian Neuroscience Institute, Nuffield Department of Clinical Neurosciences, Sir William Dunn School of Pathology, University of Oxford, OMPI G, South Parks Road, Oxford, OX1 3RE UK

**Keywords:** Melanopsin, Single-nucleotide polymorphisms, Opsins, Photopigment

## Abstract

Melanopsin is a blue light-sensitive opsin photopigment involved in a range of non-image forming behaviours, including circadian photoentrainment and the pupil light response. Many naturally occurring genetic variants exist within the human melanopsin gene (*OPN4*), yet it remains unclear how these variants affect melanopsin protein function and downstream physiological responses to light. Here, we have used bioinformatic analysis and in vitro expression systems to determine the functional phenotypes of missense human *OPN4* variants. From 1242 human *OPN4* variants collated in the NCBI Short Genetic Variation database (dbSNP), we identified 96 that lead to non-synonymous amino acid substitutions. These 96 missense mutations were screened using sequence alignment and comparative approaches to select 16 potentially deleterious variants for functional characterisation using calcium imaging of melanopsin-driven light responses in HEK293T cells. We identify several previously uncharacterised *OPN4* mutations with altered functional properties, including attenuated or abolished light responses, as well as variants demonstrating abnormal response kinetics. These data provide valuable insight into the structure–function relationships of human melanopsin, including several key functional residues of the melanopsin protein. The identification of melanopsin variants with significantly altered function may serve to detect individuals with disrupted melanopsin-based light perception, and potentially highlight those at increased risk of sleep disturbance, circadian dysfunction, and visual abnormalities.

**Electronic supplementary material:**

The online version of this article (10.1007/s00018-018-2813-0) contains supplementary material, which is available to authorized users.

## Introduction

Melanopsin (*OPN4*) is a blue light-sensitive opsin-type G-protein coupled receptor (GPCR) that is expressed within a small subset of retinal ganglion cells of the human retina [1–3], termed intrinsically photosensitive retinal ganglion cells (ipRGCs) [4–6]. These inner retina photoreceptors characteristically mediate a range of non-image forming (NIF) responses to light, including circadian photoentrainment [7–9], regulation of sleep [10–13], and the pupillary light response [14, 15]. However, recent evidence has revealed additional roles for melanopsin-based light perception in visual signalling pathways [16–19], and during development of visual [20] and non-visual systems [21]. Furthermore, the growing appreciation of how sleep and circadian disruption may contribute to the onset of symptoms of neurological and psychiatric conditions has highlighted the importance of the melanopsin system to human health and disease [22].

Like other genes, naturally occurring mutations and sequence changes are known to exist within the human melanopsin gene. The most common forms of genetic variation are single-nucleotide polymorphisms (SNPs), where an individual nucleotide base differs at a specific location within the genome in more than 1% of the population [23]. Substitutions that occur less frequently are classified as rare variants [24]. The majority of genetic variants occur in non-coding regions of genes or lead to synonymous amino acid substitutions [25]. However, genetic variation can also cause frameshifts, nonsense and missense mutations, leading to disruption or total loss of protein function and potentially causing disease. For example, mutations in rhodopsin are a leading cause of blindness, with over 150 rhodopsin mutations associated with human retinal disease [26, 27].

Both common SNPs and rare gene variants have been reported in the human melanopsin gene (*OPN4*) from whole-genome and exome sequencing projects, such as the 1000 Genomes project [28]. The *OPN4* sequence variants identified to date are collated in the NCBI Short Genetic variation database (dbSNP) [29] and number over 1200 in total. Of these, 96 *OPN4* variants reported in the dbSNP database lead to non-synonymous missense mutations, yet the functional consequences of these mutations on melanopsin activity, and the extent to which they may influence melanopsin-dependant behaviours in humans, remain largely undetermined. The exceptions are a series of studies examining the possible association of two *OPN4* SNPs, P10L and T394I, with abnormal melanopsin-driven behaviours and higher frequency of seasonal affective disorder [30–35]. However, at present, the mechanisms by which *OPN4* polymorphisms may influence melanopsin protein function are currently unclear.

Identification of *OPN4* mutations that give rise to altered melanopsin function is an important step towards understanding the role of melanopsin in human physiology and behaviour. Yet, the structure–function relationships of melanopsin are still poorly defined. The majority of targeted mutagenesis studies of melanopsin have explored post-translational modification sites [36–40] and the retinal chromophore-binding pocket [41–43]. It is, therefore, difficult to predict the functional consequences of *OPN4* variants based solely on existing knowledge of melanopsin structure–function.

All GPCRs, including melanopsin, share a common seven-transmembrane helical structure and many highly conserved functional domains [44, 45]. As such, studies of sequence homology with other GPCRs offer a valuable approach for predicting the impact of *OPN4* variants. As one of the most-well characterised GPCRs, rhodopsin is especially useful as a model for structure–function relationships in GPCRs and, in particular, other opsin photopigments. Opsin photopigments, which consist of a vitamin A-derived retinal chromophore bound to an opsin protein moiety, have a conserved activation mechanism. Isomerisation of the chromophore after absorbing a photon causes a conformation change in the opsin protein from inactive to an active state, triggering activation of a G-protein signalling pathway and downstream phototransduction cascades [46]. The critical residues responsible for these functions in rhodopsin have been extensively documented based on crystal structures (first in [47], summarised in [48]), site-directed mutagenesis studies [49–53], and the identification of over 150 rhodopsin mutations associated with retinal disease [26, 27].

This comparative approach has its limits and is only valid for *OPN4* variants that have equivalent residues within rhodopsin. While sequence homology is relatively high within transmembrane regions and other key conserved GPCR domains, overall rhodopsin and melanopsin share only 28% amino acid identity [45]. The N- and C-terminal domains of melanopsin are highly variable between different species, and melanopsin contains several extended insertions within the intracellular loops that are absent in rhodopsin. Furthermore, there are several functional differences between melanopsin and rhodopsin, including G-protein specificity ([54–57], reviewed in [58]) and chromophore regeneration mechanisms [55, 56, 59–61], suggesting that key functional residues may differ between these two opsins even within highly conserved domains. It is, therefore, necessary to directly investigate the functional properties of melanopsin genetic variants to determine their role in melanopsin activity.

Here, bioinformatic analysis of sequence alignments and comparative approaches were used to identify 16 missense *OPN4* variants likely to result in loss-of-function phenotypes from the 96 known *OPN4* missense mutations. Further functional characterisation of these 16 variants was completed using in vitro heterologous expression combined with calcium imaging of melanopsin-driven light responses. Using this approach, we identified a number of previously uncharacterised *OPN4* variants with atypical functional properties. These data provide important insights into the key functional domains of the melanopsin protein and highlight individuals with potential increased risk of visual, sleep, and circadian dysfunction.

## Materials and methods

### Identification of *OPN4* variants

Polymorphisms in the *OPN4* gene (Gene ID 94233, transcript NM_033282.2) were identified from the NCBI Short Genetic Variation database (dbSNP) Build 140 [29]. The dbSNP is a large public database that collates simple genetic variations, including common SNPs and rare genetic variants. Each genetic variant in dbSNP is assigned a reference SNP ID (rs#), and includes information on the variant position, alleles, and validation status. Missense variants were identified and prioritised for further in vitro screening using criteria shown in Table 1. Melanopsin protein sequences from the following species were used for multiple alignment—*Homo sapiens* (NP_150598.1, NP_001025186.1), *Pan troglodytes* (XP_001135445.1, XP_001135533.1), *Macaca mullata* (XP_001088248.2), *Canis familiaris* (XP_853735.2), *Bos taurus* (NP_001179328.1), *Felis catus* (AAR36861), *Mus musculus* (NP_038915.1, NP_001122071.1), *Rattus Norvegicus* (NP_620215.1), *Phosopus sungorus* (AAU11506), *Spalax ehrenbergi* (CAO02487), *Gallus gallus* (ABX10832.1, ABX10833.1, ABX10834.1, NP_989956.1, ABX10831.1), *Sminthopsis crassicaudata* (ABD38715), *Danio rerio* (NP_001245153.1, ADN39430, NP_840074.1, NP_001243006.1, ADN39434.1), *Podarcis siculus* (AAY34941.2), *Xenopus laevis* (NP_001079143.1), and *Brachiostoma belcheri* (BAE00065). Multiple sequences for a given species represent different splice variants or distinct melanopsin genes, which are duplicated in non-mammals [62]. Protein sequences were aligned using multiple sequence alignment software MAFFT [63].

**Table 1:** Criteria for identifying *OPN4* variants for in vitro screening

Variant property	Reasoning	Method	Criteria for inclusion
Validation status	dbSNP accepts submissions from a variety of sources of varying quality. Variants with multiple submissions are less likely to be false positives	Restricted analysis to “validated” variants obtained by non-computational methods and those with frequency data attached	Validated by multiple submissions, preferably including the 1000 Genomes project [28]
Conservation between OPN4 of different species	Highly conserved amino acids of OPN4 are more likely to be functionally important	Aligned 20 OPN4 amino acid sequences from 16 different species with multiple alignment using fast Fourier transform (MAFFT) software	Substitutions that occur at highly conserved OPN4 residues
Amino acid substitution properties	Substitutions that substantially change the biochemical properties of amino acids are more likely to affect protein function [64]	Compared the charge, hydrophobicity, bond formation, polarity and side chain size of amino acid substitutions using NCBI amino acid explorer [65]	Significant change in hydrophobicity, charge or polarity of amino acid
Location in protein	Mutations located in key functional domains, such as transmembrane helices or intracellular loops, are more likely to disrupt function [66]	Human OPN4 and bovine rhodopsin protein sequences were aligned and OPN4 domains defined based on rhodopsin structure [47]	Substitutions that occur in transmembrane helices or intracellular loops.
Functional role of equivalent residue in rhodopsin	Residues conserved between OPN4 and rhodopsin may perform similar functions, and show similar effects following mutation	Comparison to known rhodopsin mutations made following alignment of human OPN4 and bovine rhodopsin protein sequences	Evidence of functional effects in rhodopsin
Minor allele frequency (MAF)	The more frequent the minor allele, the greater potential relevance to the human population as a whole	Frequency data is reported in dbSNP, with MAF expressed as a value from 0 to 1	Variants with higher MAF scores (> 0.01)

A BLAST protein alignment [67] of human melanopsin (NP_150598.1) and bovine rhodopsin (NP_001014890.1) was used to define melanopsin protein domains and identify the equivalent position of the *OPN4* variants within the rhodopsin protein (Figure S1). A literature search was then performed in NCBI PubMed (http://www.ncbi.nlm.nih.gov/pubmed/) and Google Scholar (http://www.scholar.google.co.uk/) to determine whether the equivalent residues are functionally significant in rhodopsin, based on analysis of crystal structure [47, 68, 69] and lists of rhodopsin mutations known to be associated with retinitis pigmentosa [26, 70].

### Generation of *OPN4* expression vectors

Single-nucleotide point mutations were introduced to pcDNA3.1 plasmid (Invitrogen) containing human melanopsin (NM_033282.2) with a 1D4 tag [57] using a Quikchange II XL site-directed mutagenesis kit (Stratagene). Mutagenesis primers are shown in Table S2. Successful introduction of mutations was confirmed by Sanger sequencing (Source Biosciences). Plasmid production and purification was performed using standard techniques.

### Cell culture and transient transfection

HEK293T cells (ATCC) were cultured in DMEM (Sigma) with 10% foetal bovine serum (Life Technologies), 2 mM l-glutamine (Sigma), and 1% (v/v) penicillin/streptomycin (Sigma). Cells were maintained in a humidified incubator at 37 °C with 5% CO_2_, fed fresh media every 2–3 days and passaged before reaching confluence. 24 h after seeding into multi-well plates (see below), cells were placed in antibiotic-free DMEM with 10% foetal bovine serum and 2 mM l-glutamine, and transiently transfected using Genejuice transfection reagent (Merck Millipore) according to the manufacturer’s instructions with a 1:3 ratio of DNA (µg) to Genejuice (µl).

### Immunocytochemistry

HEK293T cells were seeded into 6-well plates containing 13 mm glass coverslips at ~ 1 × 10^5^ cells per well and transfected as described above. 48 h after transfection, cells were fixed with 4% methanol-free paraformaldehyde (Thermo Scientific) in phosphate buffered saline (PBS) for 10 min at room temperature and immunostained as described previously [71]. A rabbit polyclonal anti-human melanopsin antibody (H-300, Santa Cruz Biotechnology, 1:400) was incubated for 1 h at room temperature. Secondary antibody was donkey anti-rabbit IgG conjugated to Alexa 568 (Life Technologies, 1:200). After final wash step, nuclear counterstaining was performed with 0.5 μg/ml DAPI for 10 min at room temperature. Coverslips were then mounted onto glass microscope slides using Prolong Gold anti-fade mounting media (Life Technologies). Fluorescence images were collected using an inverted LSM 710 laser scanning confocal microscope (Zeiss) and Zen 2009 image acquisition software (Zeiss). Individual channels were collected sequentially. Laser lines for excitation were 405 and 561 nm, with emissions collected between 440–480 and 580–625 nm for blue and red fluorescence respectively. Images were collected using  a 40× objective with images collected every 1.0 µm in the *Z* axis. Global enhancement of brightness and contrast was performed using ZenLite 2011 software (Zeiss) and applied equally to all images.

### Fluorescent calcium imaging

HEK293T cells were seeded into 24-well plates at 4 × 10^4^ cells per well and transfected with pcDNA3.1 *OPN4*-1D4 plasmid 24 h later. 24 h after transfection, cells were incubated with 5 µM Fluo-4 AM calcium indicator dye (Life Technologies), 0.015% Pluronic F-127 (Life Technologies), 2.5 µM probenecid (Life Technologies), and 20 µM 9-*cis* retinal (Sigma) for 20 min at 37 °C. Following dye loading, cells were incubated for a further 10 min at 37 °C in DMEM with 2.7 µM probenecid and then transferred to Hank’s buffered saline solution without additional calcium (Gibco) prior to calcium imaging. All steps were conducted under dim red light (610 ± 10 nm, 3.02 × 10^11^ photons/cm^2^/s in the working area). Calcium imaging was performed using a FLUOstar Omega plate reader (BMG Labtech) at room temperature. Total fluorescence values from each well were collected every 2 s for a total of 120 s, with individual wells imaged sequentially. Each data point was generated by averaging fluorescence values collected from 200 repeated 5 ms flashes of light generated by the plate reader’s internal xenon flash bulb with excitation and emission filters of 485 nm (12 nm bandwidth) and 520 nm (30 nm bandwidth), respectively. These multiple flashes of excitation light resulted in a near continuous illumination of cells (485 ± 6 nm, 1.21 × 10^14^ photons/cm^2^/s) and were sufficient to activate the melanopsin photopigment without further light stimulation.

Each 24-well plate contained four technical replicates of five different *OPN4* variants and wild-type *OPN4* positive control. Values for the four technical replicates of each plasmid obtained from individual plates were averaged to produce a single biological replicate for each sample. A total of six biological replicates (each the result of a separate transfection) were performed for each *OPN4* variant or control. For analysis, biological replicates were first normalised to baseline (first recorded fluorescence value) for response amplitude comparisons (Baseline = 0, Δ*F*/*F*_*0*_), then normalised to maximum for comparing response kinetics (Baseline = 0, maximum = 1, Δ*F*/*F*_max_). As no statistical difference was observed in response properties of *OPN4* WT replicates from different plates (Figure S2), the *OPN4* variants were compared to pooled *OPN4* WT data for subsequent comparisons.

### Statistical analysis

Statistical tests were conducted using SPSS 22.0 (IBM). Unless stated otherwise, all comparisons between groups were tested using one-way between-subjects analysis of variance (ANOVA) with genotype as the independent variable. For one-way ANOVA, a post hoc Dunnett’s test was used to explore significant main effects of genotype, using *OPN4* WT as the control group. A significance threshold of *p* ≤ 0.05 was used. In all figures, asterisk (*) indicates *p* < 0.05, **p < 0.01, and ***p < 0.001. Error bars show standard error of mean. Where error bars are smaller than data symbols, error bars are not shown.

## Results

### Identification and selection of 16 *OPN4* variants for in vitro screening

**Fig. 1:**
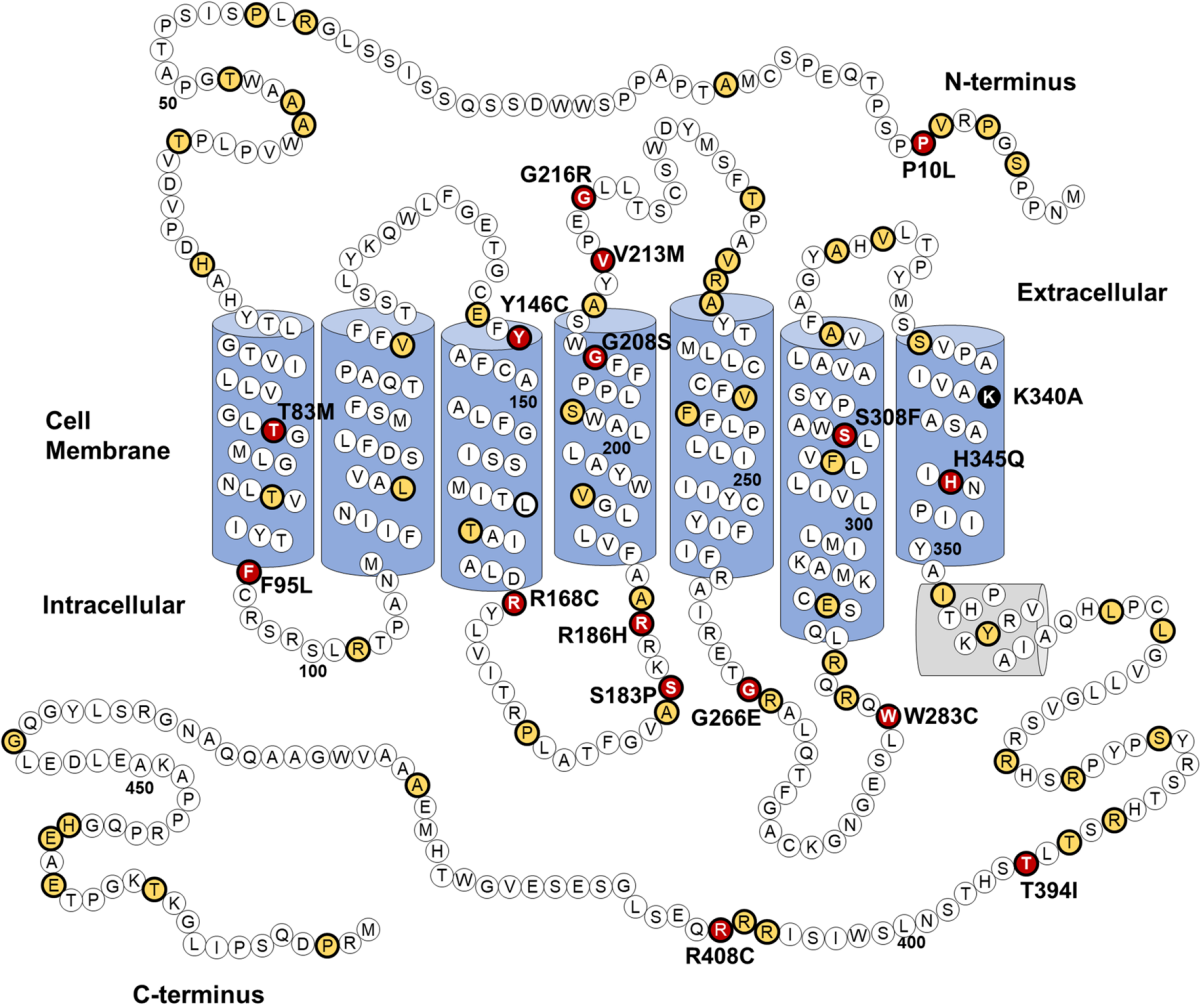
*OPN4* genetic variants selected for in vitro screening. Location of 96 naturally occurring non-synonymous amino acid substitutions (thick black outline) in the human OPN4 protein, of which 16 (red) were screened using immunocytochemistry and calcium imaging of melanopsin-driven light responses. Location of variants not screened in vitro (yellow) and the K340A control mutation (black) is also shown. Secondary structure based on homology with bovine rhodopsin [47]

At the time this study was conducted 1242 genetic variants of the *OPN4* gene were identified in the dbSNP database (build 140), of which 96 lead to non-synonymous amino acid substitutions. A detailed description of these 96 *OPN4* missense mutants is provided in Supplementary Table 1. Using sequence alignment and comparative approaches a subset of these 96 *OPN4* variants were prioritised for functional screening in vitro using six main selection criteria, including validation status, conservation of residues in melanopsin across 16 different species, biochemical properties of the amino acid substitution, location of the mutation within key functional domains, functional role of the equivalent residue in rhodopsin (if present), and the overall frequency of the variant within the population. A detailed description of the criteria and methods used for selecting variants is shown in Table 1. *OPN4* variants did not have to meet all criteria to be included for further analysis, with preference given to variants with altered amino acid biochemical properties and those occurring at sites highly conserved amongst melanopsin sequences of different species. Based on the selection criteria, 16 *OPN4* variants were classified as being likely to result in change of function phenotypes and were selected for further in vitro functional testing. The location of the 16 *OPN4* variants within the human melanopsin protein is shown in Fig. 1. Details of how each variant matched the criteria for selection is outlined in Table 2.

**Table 2:** Properties of *OPN4* variants selected for in vitro screening

Mutation	Conservation between species	Amino acid properties	Protein position	MAF	Equivalent rhodopsin residue	Functional role of equivalent residue in rhodopsin
P10L^a^ rs2675703	Not conserved	Both non-polar and hydrophobic	NT	**0.08 (T)**	Not conserved	
T83M rs202029105	Conserved in *Opn4M,* l/V/L in *Opn4X*	**Polar to non-polar**	**TM1**	0.0002 (T)	L50	Part of chromophore channel [72]
F95L rs573102269	**Conserved in all species**	Loss of large side chain. Both hydrophobic and non-polar	**TM1**	0.0002 (C)	T62	H-bond between TM1 and TM2 [73]
Y146C rs200099863	**Conserved in all species**	Both polar and similar hydrophobicity	**TM3**	0.0002 (G)	**E113**	**Schiff Base Counterion [** 49 **]**
R168C^b^ rs143641898	**Conserved in all species**	**Positive charge to polar. Increased hydrophobicity**	**TM3 / IL2**	0.0005 (T)	**R135** ^**c**^	**Conserved DRY motif [** 47 **]**
S183P rs151123640	**Conserved in most species (T in Amphioxus)**	**Polar to non-polar**	IL2	0.0002 (C)	E150^c^	Required for normal membrane trafficking [74]
R186H^b^ rs141089672	Conserved in most species (K in Chicken and Zebrafish)	**Both positive charge, increase in hydrophobicity**	IL2	0.0005 (A)	Not conserved	
G208S rs549998450	**Conserved in all species**	**Non-polar to polar. Introduce large side chain**	**TM4 EL2**	0.0004 (A)	**G174** ^**c**^	Unknown
V213M rs202171086	**Conserved in** ***Opn4M,*** **l/V in** ***Opn4X***	Both hydrophobic and non polar	EL2	0.0002 (A)	**I179**	Unknown
G216R rs201432667	**Conserved in all species**	**Non-polar to polar, loss of hydrophobicity**	EL2	0.0002 (A)	**G182** ^**c**^	**Part of H-bond within EL2 [** 75 **]**
G266E rs576858032	Conserved in mammals, S/G/N in non-mammals	**Non-polar to negative charge. Introduce large side chain and decrease hydrophobicity**	IL3	0.0002 (A)	A234	Arrestin Binding Site [76]
W283C rs145634412	Not conserved	**Non-polar to polar, loss of large side chain**	IL3	0.001 (T)	Not Conserved	
S308F rs559392371	Conserved in all species	**Positive charge to polar. Similar hydrophobicity**	**TM6**	0.001 (T)	**C264**	**Part of xWxPY motif** [77, 78]
H345Q rs184720512	Conserved in *Opn4M,* Y in *Opn4X*	**Polar to non-polar, increase hydrophobicity**	**TM7**	0.0002 (G)	Y301	H-bond between TM2 and 7 [73]
T394I^a^ rs1079610	**Conserved as S or T in all species**	**Polar to non-polar, increase hydrophobicity**	CT	**0.25 (C)**	Not Conserved	
R408C rs199878852	Conserved in mammals, not conserved in non-mammals	**Positive charge to polar**	CT	0.0002 (T)	Not Conserved	

Highlighted in bold are properties of each variant that met criteria for further investigation. Minor allele is shown in brackets beneath MAF score. All variants were validated by multiple submissions and 1000 Genomes project [28], unless stated otherwise

^a^Variants with published data

^b^Variants validated by multiple submissions only

^c^Equivalent residue in rhodopsin is mutated in individuals with retinitis pigmentosa [70]

Overall, only five of the 96 non-synonymous mutations identified, P10L, T394I, G444D, R406W, and L365V, occur in more than 1% of the population and can be considered polymorphic. SNPs G444D, R406W, and L365V are located in poorly conserved domains of the melanopsin protein and produce only minor changes in biochemical properties of amino acids (Fig. 1). These substitutions were, therefore, deemed unlikely to significantly affect melanopsin function and were excluded from further analysis. Indeed, the majority of missense variants identified were rejected due to their location at sites poorly conserved amongst melanopsins, suggesting a non-critical role in melanopsin function. Given the suggested impact of *OPN4* SNPs P10L and T394I on human behaviour [30–35] and their comparatively high minor allele frequency (MAF), both P10L and T394I were selected for further in vitro functional testing.

Notably, we identified several *OPN4* variants that occur at highly conserved sites known to be critical for normal rhodopsin and GPCR function, including the conserved E/DRY motif required for G-protein activation [79], the vertebrate opsin counterion in transmembrane helix 3 which maintains stability of the chromophore when bound to opsin [49], and the conserved xWxPY opsin motif in transmembrane helix 6 [77, 78], which is essential for the opsin confirmation change necessary to trigger the phototransduction cascade. Mutation of these highly conserved sites severely disrupts protein function [49, 80, 81] and may, therefore, result in similar loss-of-function phenotypes in melanopsin. The minor allele frequency of variants located within these highly conserved sites indicates that these potentially deleterious mutations are rare (MAF < 0.01, Table 2).

We also identified a number of *OPN4* variants located in the less conserved regions of the N-terminus, C-terminus, and intracellular loops of melanopsin, for which there are no homologous residues in rhodopsin—which may represent variants at sites with melanopsin-specific functional properties. These included P10L, R186H, W283C, T394I, and R408C. In addition to the 16 *OPN4* variants selected for in vitro screening, the *OPN4* K340A mutant was generated to act as a negative control during calcium imaging assays. This construct contains a targeted site-specific mutation (i.e., one not found naturally in the human genome) at the retinal chromophore-binding site rendering melanopsin non-functional [41, 42].

### Cellular localisation and membrane trafficking of mutant OPN4 protein

**Fig. 2:**
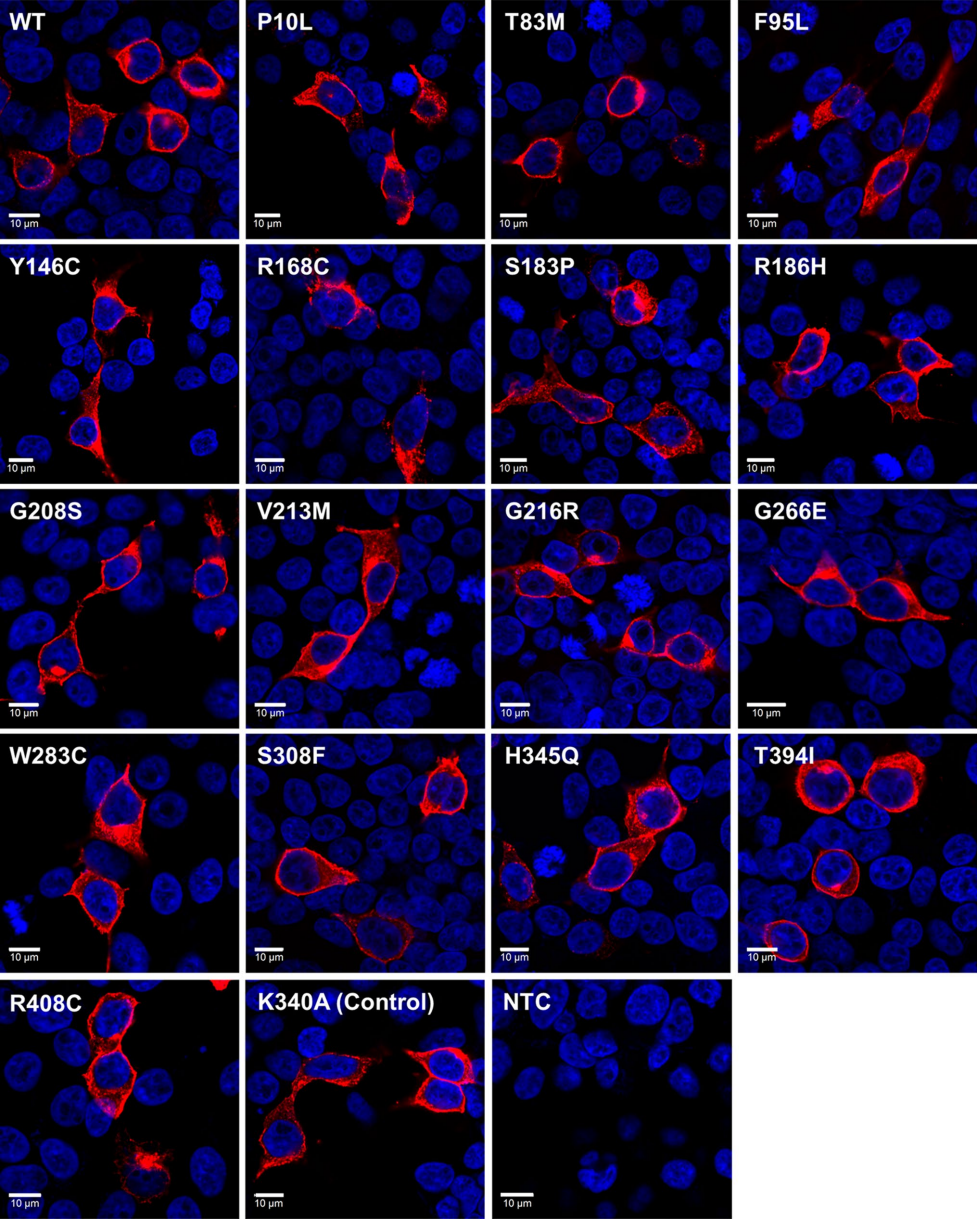
Melanopsin variants show normal membrane localisation. Heterologous expression of pcDNA3.1 *OPN4* WT and *OPN4* variants in HEK293T cells labelled with anti-OPN4 antibody (red) and DAPI nuclear stain (blue). Scale bar 10 µm

In the first instance, the subcellular localisation and membrane trafficking of selected *OPN4* variants was examined using immunocytochemistry following transient transfection in HEK293T cells (Fig. 2). Overall, levels of OPN4 protein staining were similar between the different variants. All variants appeared to be trafficked normally and were expressed at the plasma membrane, with no obvious differences observed in overall levels of expression or cellular localisation between *OPN4* WT and any *OPN4* variants examined. Notably, we did not observe any evidence of increased protein aggregation in the endoplasmic reticulum for any *OPN4* variant tested, as may be expected for mutations affecting tertiary structure and protein folding [26, 82].

### Functional characterisation of *OPN4* variants using fluorescent calcium imaging

**Fig. 3:**
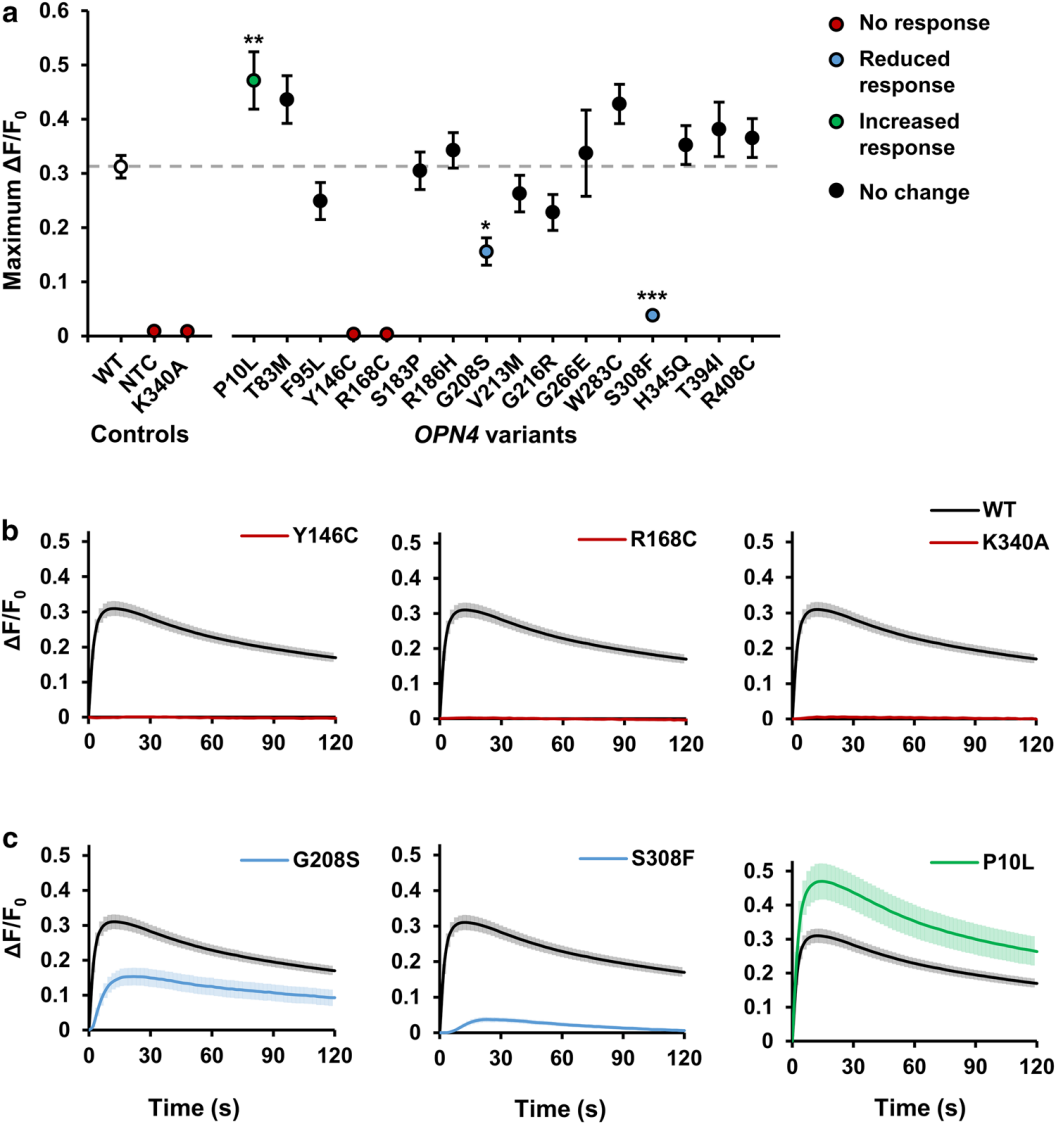
Melanopsin variants show abnormal intracellular calcium responses to light. Intracellular calcium levels of HEK293T cells transiently transfected with pcDNA3.1 *OPN4* WT and *OPN4* variants were monitored using the fluorescent calcium indicator Fluo4-AM. Melanopsin-driven light responses were triggered by the first light exposure used for fluorescent imaging (485 ± 6 nm). **a** Mean response amplitude (maximum Δ*F*/*F*_*0*_) for *OPN4* WT and each *OPN4* variant tested. Dashed grey line shows *OPN4* WT response. **b**, **c** Traces showing the kinetics of intracellular calcium responses recorded from **b** non-functional *OPN4* variants (red) and **c** *OPN4* variants with significantly attenuated (blue) or elevated (green) intracellular calcium responses compared to *OPN4* WT (black). *N* = 6 biological replicates for all groups except *OPN4* WT (*N* = 24). Asterisk indicates significant Dunnett’s post hoc test (*p* > 0.05) compared to *OPN4* WT. NTC is no transfection control. Error bars show standard error of mean. Where error bars are smaller than symbol, error bars are not shown

We next used in vitro fluorescent calcium imaging methods to characterise the functional properties of each of the 16 selected *OPN4* variants. In line with the previous reports, light stimulation (485 ± 6 nm, 1.21 × 10^14^ photons/cm^2^/s) led to a rapid elevation of intracellular calcium levels within HEK293T cells transfected with *OPN4* WT (Δ*F*/*F*_0_, Fig. 3) [37, 38, 57], consistent with the activation of a Gα_q/11_ signalling cascade and release of calcium from intracellular stores [41, 42, 83].

Several *OPN4* variants failed to show any detectable changes in intracellular calcium levels in response to light (Fig. 3a, b). This included the negative control mutation K340A, which is incapable of binding retinal chromophore [41, 42], but also *OPN4* variants Y146C and R168C. The response amplitudes of these variants were indistinguishable from untransfected cells, *F*(2,15) = 2.31, *p* = 0.134 (One-way ANOVA), suggesting that these mutations render *OPN4* protein non-functional.

The remaining *OPN4* variants appeared to produce functional *melanopsin* protein capable of coupling to a Gα_q/11_ signalling pathway, as shown by light-induced elevations of intracellular calcium levels (Fig. 3a). Whilst the majority of *OPN4* variants showed response amplitudes similar to that observed for *OPN4* WT controls, there was a significant main effect of genotype on response amplitude for functional variants, *F*(14, 93) = 7.44, *p* < 0.001 (One-way ANOVA). A Dunnett’s post hoc test revealed this was driven by three *OPN4* variants with significantly attenuated or elevated responses compared to *OPN4* WT (Fig. 3c). Both *OPN4* S308F (*p* < 0.001) and G208S (*p* = 0.011) showed a significant reduction in response amplitude compared to *OPN4* WT, with this effect observed consistently across all biological replicates. Only one variant, SNP P10L, demonstrated an overall elevation of response amplitude (*p* = 0.009). However, the increased response of P10L relative to *OPN4* WT was not consistently observed across all biological replicates.

**Fig. 4:**
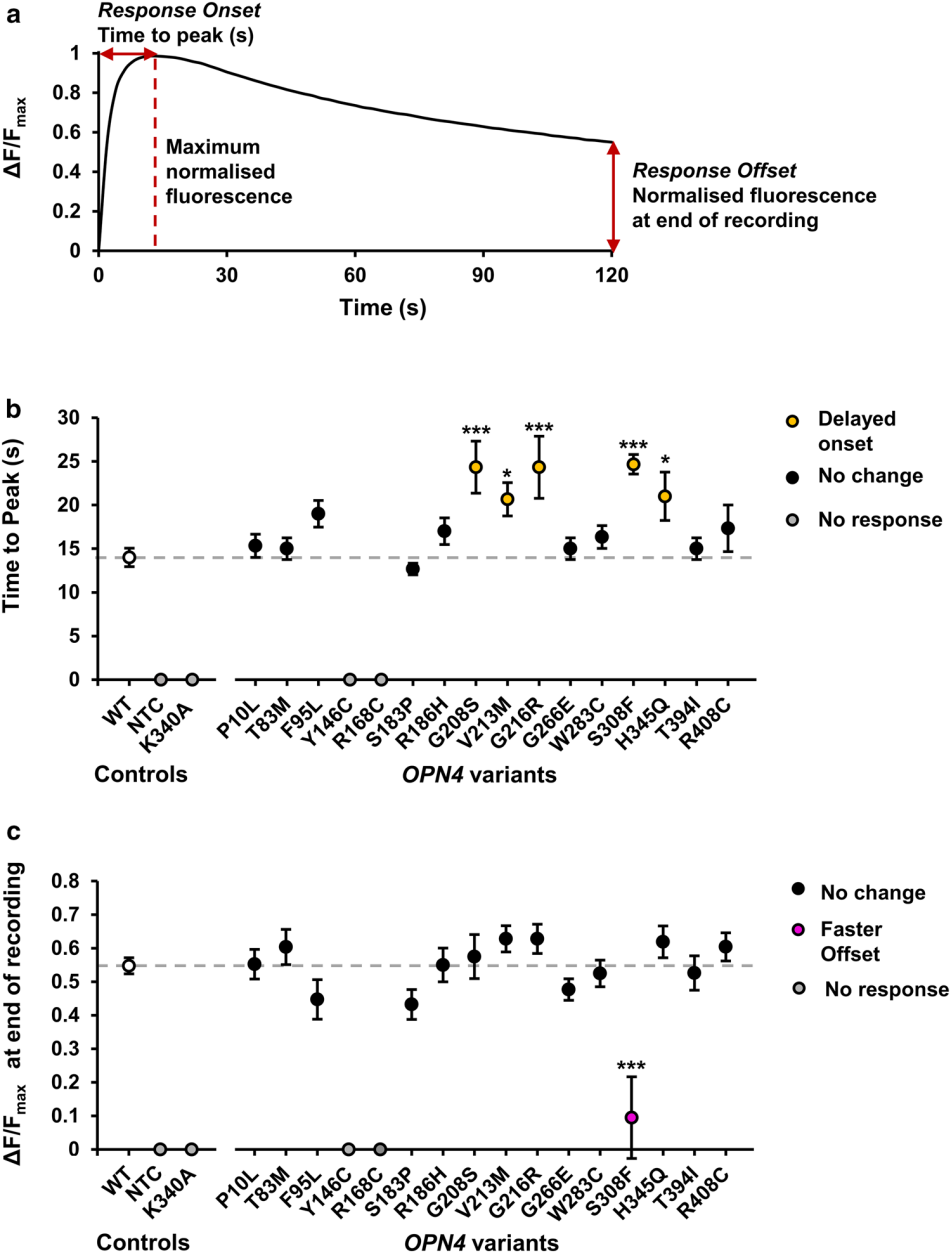
*OPN4* variants have abnormal response kinetics. **a** Parameters used to define response kinetics. Data were first normalised to baseline (Baseline = 0, Δ*F*/*F*_*0*_), then normalised to maximum for comparing response kinetics (Baseline = 0, maximum = 1, Δ*F*/*F*_max_). Response onset was measured as time to peak fluorescence (s). Response offset was measured as relative fluorescence recorded at end of recording (120 s after the first light exposure). **b** Mean response onset for each *OPN4* variant tested. **c** Mean response offset for each *OPN4* variant tested. Dashed grey line shows *OPN4* WT response. *N* = 6 biological replicates for all *OPN4* variants and *N* = 24 biological replicates for *OPN4* WT control. Asterisk and triple asterisk represent significant post hoc Dunnett’s tests (*p* < 0.05 and *p* < 0.001 respectively) compared to *OPN4* WT. NTC is no tranfection control. Error bars show standard error of the mean. Where error bars are smaller than symbol, error bars are not shown

Comparison of response kinetics identified several *OPN4* variants with modified response properties (Fig. 4). There was a significant main effect of genotype on rate of response onset, measured as time to peak fluorescence, *F*(14,93) = 4.73, *p* < 0.001 (One-way ANOVA), which a Dunnett’s test revealed was due to slower response onset in five *OPN4* variants, G208S (*p* < 0.001), G216R (*p* < 0.001), S308F (*p* < 0.001), V213 M (*p* = 0.038), and H345Q (*p* = 0.025) (Fig. 4b). As a measure of response decay, normalised fluorescence values were compared at the end of the recordings (120 s after first light exposure). Only a single *OPN4* variant, S308F, demonstrated a faster response offset compared to *OPN4* WT controls (*p* < 0.001) (Fig. 4c) as determined using a Dunnett’s post hoc test to explore the significant main effect of genotype on response decay, *F*(14, 93) = 5.73, *p* < 0.0001 (one-way ANOVA). A summary of the functional phenotypes observed from all *OPN4* variants tested in vitro is shown in Table 3.

**Table 3:** Summary of key features and phenotype of 16 screened *OPN4* variants

Variant	Key features	Observed phenotype (relative to *OPN4* WT)		
**Normal**				
T83M	Change in polarity	No difference		
F95L	Conserved in all species	No difference		
S183P	Conserved in most species change in polarity	No difference		
R186H	Change in hydrophobicity	No difference		
G266E	Change in charge and hydrophobicity	No difference		
W283C	Change in polarity	No difference		
T394I	SNP associated with abnormal NIF behaviour in humans. Conserved in all species as S/T	No difference		
R408C	Change in charge	No difference		
**Non-functional**				
Y146C	Conserved in all species	No response		
R168C	Conserved in all GPCRs. Change in charge and hydrophobicity	No response		
**Abnormal function**		**Amplitude**	**Onset**	**Offset**
P10L	SNP associated with abnormal NIF behaviour in humans.	Elevated	No difference	No difference
G208S	Conserved in all species change in polarity	Attenuated	Delayed	No difference
V213M	Conserved in all species as l/V	No difference	Delayed	No difference
G216R	Conserved in all species change in polarity and hydrophobicity	No difference	Delayed	No difference
S308F	Conserved in all species change in charge	Attenuated	Delayed	Faster
H345Q	Change in polarity and hydrophobicity	No difference	Delayed	No difference

## Discussion

Here, we show that bioinformatic approaches using sequence alignment combined with in vitro calcium imaging provide a high-throughput method for identifying non-synonymous human *OPN4* mutations which result in altered melanopsin signalling. By focusing on variants located at highly conserved sites or those leading to a significant change in amino acid biochemical properties, we prioritised 16 of 96 recorded missense *OPN4* variants for in vitro screening. Immunocytochemistry demonstrated that all 16 melanopsin variants were successfully trafficked to the plasma membrane, while assessment of melanopsin-driven calcium responses identified several variants with abnormal responses to light.

### Identification of *OPN4* variants with abnormal functional phenotypes

Of the seven variants that showed abnormal calcium responses, four are particularly strong candidates for key functional residues in the melanopsin protein. Introduction of mutations Y146C and R168C in OPN4 abolished responses to light, and two further variants, S308F and G208S reliably demonstrated attenuated response amplitude and delayed onset compared to OPN4 WT controls. Furthermore, these deleterious variants occur at sites highly conserved between different classes of opsin (and GPCRs in general), providing likely mechanisms by which these mutations cause loss-of-function. For example, R168C is part of the E/DRY motif found in nearly all GPCRs. The “ionic lock” between these residues and a conserved negative-changed amino acid in transmembrane helix 6 (TM6) is necessary for maintaining the inactive conformation of GPCRs [47, 77, 79, 80]. S308F is part of the xWxPY motif located within TM6, acting as a “rotamer toggle switch” [77, 84], which facilitates rotation of TM6 into the active confirmation following photoisomerisation of retinal chromophore [77, 85]. Given that variation at these sites attenuates or abolishes melanopsin activity, it is highly likely that these motifs perform similar functions in melanopsin.

A third deleterious variant, Y146C, occurs at a conserved opsin site in TM3. In vertebrate opsins, this site represents the glutamic acid counterion, a negatively charged amino acid that counteracts the positive charge of the Schiff base that binds the chromophore to the opsin apoprotein allowing absorption of photons in the visible spectrum [49, 86]. In invertebrate opsins, this site is occupied by a conserved tyrosine, and the glutamic acid counterion is displaced to extracellular loop 2 [87]. Interestingly, melanopsin possesses an invertebrate-like conformation of residues at these two positions. It is, therefore, surprising that variation at the vertebrate counterion location causes a total loss of melanopsin function. This site cannot act as the melanopsin counterion as tyrosine is not negatively charged; however, it is possible that this site still critically interacts with the Schiff base and disrupts retinal binding or isomerisation when mutated.

### Human *OPN4* variants inform melanopsin structure–function relationships

Overall, we find that the most damaging *OPN4* variants occur at highly conserved residues in the opsin chromophore-binding pocket. In contrast, substitutions in more variable protein domains have no observable effect even if they substantially alter hydrophobicity or charge of amino acid residues, such as R186H and G266E located in intracellular loops 2 and 3 respectively. Notably, none of the critical residues identified in the in vitro screen appear specific to melanopsin. In fact, of the five *OPN4* variants tested for which there is no equivalent residue in rhodopsin, none appear to be important for normal melanopsin function.

Interestingly, neither *OPN4* T394I nor P10L, two SNPs previously associated with abnormal and attenuated NIF behaviour in humans [30–35], showed melanopsin loss-of-function in vitro. Instead, we observed no difference between T394I and WT. Overall, we found significantly larger calcium responses for P10L compared to *OPN4* WT, although this was not consistent between all biological replicates. Based on its position in the N-terminus and the loss of a proline, there has been some speculation that P10L has a similar effect on melanopsin as the P23H mutation in rhodopsin, which leads to severe protein misfolding [82, 88]. However, our cellular localisation data suggest this is not the case, with no evidence of protein misfolding observed for P10L. As such, there is currently no clear mechanism to explain how P10L might be associated with an increased risk of seasonal affective disorder as previously reported [33–35].

It remains possible that the T394I or P10L SNPs, as well as other *OPN4* variants, may cause subtle phenotypes not captured by the in vitro calcium imaging screen used in this study. More detailed analysis using a range of light intensities and or stimulation protocols may reveal further phenotypes, such as reduced activation thresholds, altered responses to dim light, or changes in maximal responses elicited by saturating levels of light. However, it is also clear that in vitro cell line expression systems do not fully replicate the cellular environment of ipRGCs [58] and may, therefore, limit the study of *OPN4* variants with roles in ipRGC-specific aspects of melanopsin protein function, such as regulatory motifs or post-translational modification sites. Examining the function of *OPN4* variants following targeted expression into ipRGCs of the mouse retina, potentially via AAV delivery and Cre-lox based approaches [39, 40], would more closely replicate the native signalling environment of melanopsin proteins and permit in-depth functional analysis of melanopsin-specific functions, such as temporal integration, chromophore bistability, or contribution to multiple active states [60]. Nevertheless, functional characterisation of melanopsin variants in vivo is both time-consuming and expensive, and is, therefore, not suitable for screening large numbers of variants. The in vitro approach used here provides a relatively high-throughput and effective method for preliminary screening to identify *OPN4* SNP variants with altered functional properties.

### Implications of *OPN4* variants for the human population

The identification of naturally occurring *OPN4* missense mutations resulting in deleterious functional properties suggests that individuals with non-functional or severely impaired melanopsin activity may exist within the human population. Given the frequency of these deleterious variants, it would seem that individuals homozygous for these mutations (or carrying copies of two different deleterious variants) will be extremely rare. These observations alone suggest that a strong selective pressure exists to maintain normal melanopsin function. Therefore, an important consideration is whether humans either heterozygous or homozygous for these mutations will exhibit significant disruption of melanopsin-dependant behaviours. Screening such individuals for circadian disruption or abnormal non-image forming responses to light could help to determine the role of melanopsin in human physiology, and whether altered melanopsin light perception is associated with human disease.

However, based on our current understanding of the melanopsin system and studies of mice with genetically ablated melanopsin [7–14], we may expect that even highly deleterious *OPN4* variants will only result in minimal behavioural phenotypes. In the healthy retina, melanopsin-expressing ipRGCs receive synaptic input from rods and cones and act as a conduit for passing outer retina light signals to NIF centres [89]. The overlapping roles of rod, cone, and melanopsin-driven light responses in ipRGC function likely minimises the behavioural effect of melanopsin missense mutations. For example, previous reports have demonstrated that the NIF responses to light of melanopsin knockout mice are typically attenuated, but not abolished—with modest deficits typically observed only under bright light conditions. However, it has also become increasingly clear that melanopsin provides a greater contribution to ipRGC function and NIF responses to light under more physiological conditions, including control of steady-state pupil size under constant environmental illumination [90].

Recent evidence has revealed additional roles for melanopsin within the classical visual system [16–20], with melanopsin-driven light responses contributing to brightness discrimination [91, 92], contrast sensitivity [17, 18], and adaptation of visual responses [93, 94], via both retrograde signalling within the retina and modulation of dopamine signalling pathways [19, 95–99], as well as through direct projections of ipRGCs to visual centres [16, 17]. Furthermore, people with abnormal melanopsin function could exhibit other symptoms related to the role of melanopsin during postnatal development, including the patterning of retina vasculature [100], and the refinement and segregation of retinogeniculate projections to visual areas of the brain [20, 21], potentially influencing visual acuity in adulthood [21]. It is, therefore, possible that humans possessing deleterious *OPN4* variants may experience a range of behavioural symptoms related to abnormal melanopsin function, including circadian disruption, attenuated pupil constriction, reductions in contrast sensitivity, and attenuated adaptation of visual signalling pathways to changes in environmental light conditions.

## Conclusion

In conclusion, we have identified several previously uncharacterised naturally occurring missense variants in the human *OPN4* gene that result in melanopsin proteins with a significant loss-of-function phenotype. These data indicate that individuals with abnormal or abolished melanopsin activity may exist within the human population, and although rare, may be at increased risk of sleep and circadian disruption, as well as potential visual deficits. The growing availability of next-generation sequencing data is likely to lead to the identification of further rare genetic variants of the human *OPN4* gene. The methods described here for identification and in vitro functional characterisation of deleterious *OPN4* variants will be highly applicable for future investigations of *OPN4* mutations and defining structure–function relationships of the melanopsin protein.

### Electronic supplementary material

Below is the link to the electronic supplementary material.

Data S1. Properties of 96 missense mutations in the *OPN4* gene identified from dbSNP (Build 140). (XLSX 15 kb)

Figure S1. Amino acid alignment of human melanopsin (OPN4) and bovine rhodopsin (RHO). Protein domain boundaries (shown as vertical lines) are defined based on crystal structure of bovine rhodopsin [47]. TM = transmembrane helix, IL = intracellular loop, EL = extracellular loop. Highly variable N and C termini are excluded from the alignment. *OPN4* variants selected for *in vitro* screening with equivalent rhodopsin residues are highlighted in yellow. Numbers show position of the first and last residues of each row (TIF 693 kb)

Figure S2. Comparison of *OPN4* WT positive controls between plates confirms melanopsin-driven calcium responses are replicable. Intracellular calcium levels of HEK293T cells transiently transfected with pcDNA3.1 *OPN4* WT as monitored using Fluo4-AM. (a) Kinetics of intracellular calcium normalised to baseline (first data point, ΔF/F_0_). (b) Mean response amplitude, measured as maximum ΔF/F_0_, is not significantly different between biological replicates, F(3,20) = 0.54, p = 0.659 (one-way ANOVA) (c-d) Kinetics of intracellular calcium normalised to baseline, then maximum (Baseline = 0, Maximum = 1, ΔF/F_max_) are not significantly different between biological replicates, assessed using (c) Mean time to peak ΔF/F_max_, F(3,20) = 0.79, p = 0.513 (one-way ANOVA) and (d) Mean ΔF/F_max_ at end of recording, F(3,20) = 1.00, p =0.412 (one-way ANOVA). N = 6 biological replicates for each *OPN4* WT plate. n.s. = not significant. Error bars = standard error of the mean (TIF 662 kb)

Table S1. Site-directed mutagenesis primers used for construction of *OPN4* variant expression constructs. Base pair change from template sequence is shown in bold and underlined. (TIF 866 kb)
