# Supplementary figures and images for: Functional characterisation of naturally occurring mutations in human melanopsin
Source: Cell Mol Life Sci. 2018 Apr 26;75(19):3609–24. doi: 10.1007/s00018-018-2813-0 (PMC6133154; doi:10.1007/s00018-018-2813-0)

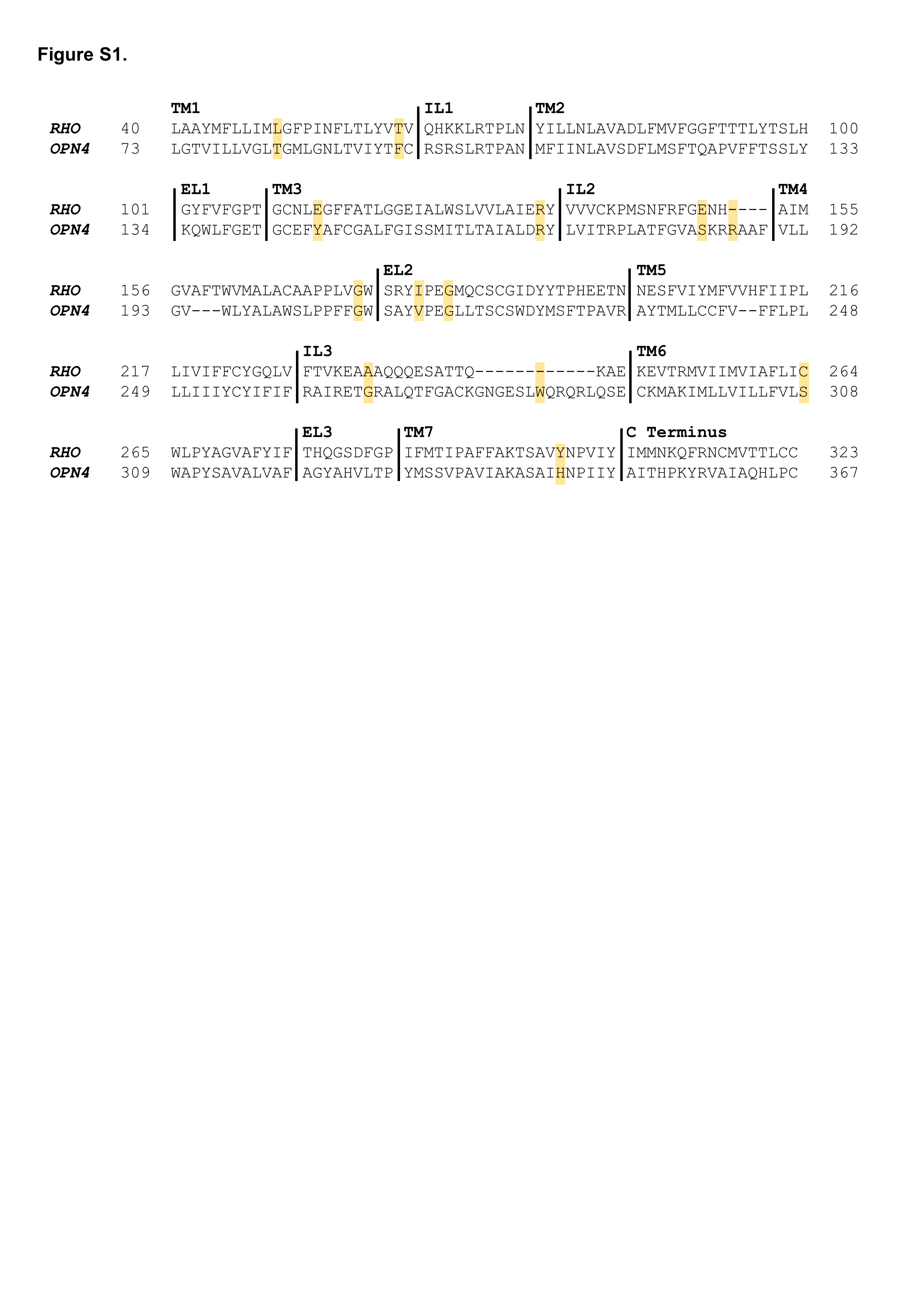

Supplement: Supplementary file 2 — Figure S1. Amino acid alignment of human melanopsin (OPN4) and bovine rhodopsin (RHO). Protein domain boundaries (shown as vertical lines) are defined based on crystal structure of bovine rhodopsin [47]. TM = transmembrane helix, IL = intracellular loop, EL = extracellular loop. Highly variable N and C termini are excluded from the alignment. OPN4 variants selected for in vitro screening with equivalent rhodopsin residues are highlighted in yellow. Numbers show position of the first and last residues of each row (TIF 693 kb) [file 18_2018_2813_MOESM2_ESM.tif]

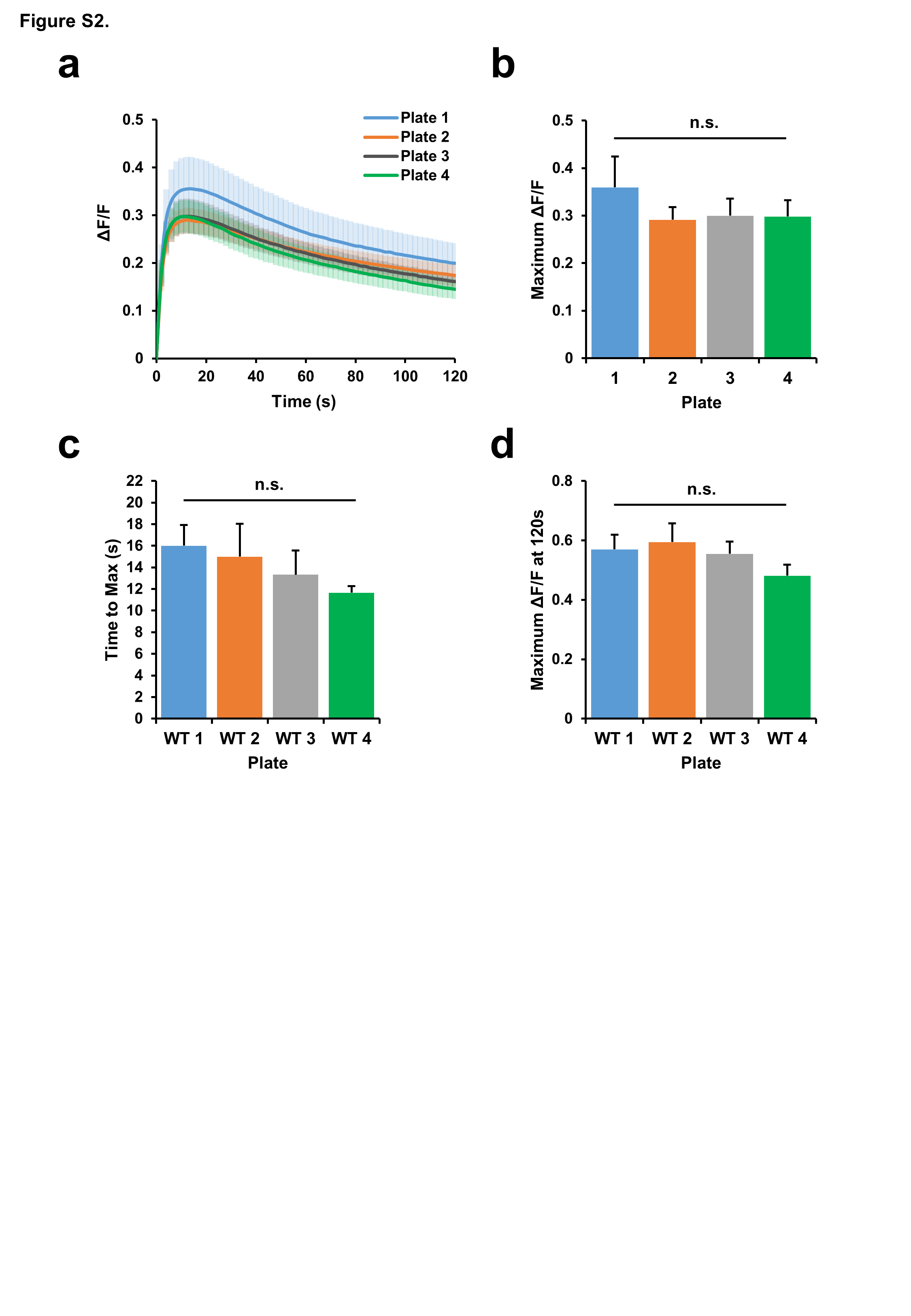

Supplement: Supplementary file 3 — Figure S2. Comparison of OPN4 WT positive controls between plates confirms melanopsin-driven calcium responses are replicable. Intracellular calcium levels of HEK293T cells transiently transfected with pcDNA3.1 OPN4 WT as monitored using Fluo4-AM. (a) Kinetics of intracellular calcium normalised to baseline (first data point, ΔF/F0). (b) Mean response amplitude, measured as maximum ΔF/F0, is not significantly different between biological replicates, F(3,20) = 0.54, p = 0.659 (one-way ANOVA) (c-d) Kinetics of intracellular calcium normalised to baseline, then maximum (Baseline = 0, Maximum = 1, ΔF/Fmax) are not significantly different between biological replicates, assessed using (c) Mean time to peak ΔF/Fmax, F(3,20) = 0.79, p = 0.513 (one-way ANOVA) and (d) Mean ΔF/Fmax at end of recording, F(3,20) = 1.00, p =0.412 (one-way ANOVA). N = 6 biological replicates for each OPN4 WT plate. n.s. = not significant. Error bars = standard error of the mean (TIF 662 kb) [file 18_2018_2813_MOESM3_ESM.tif]

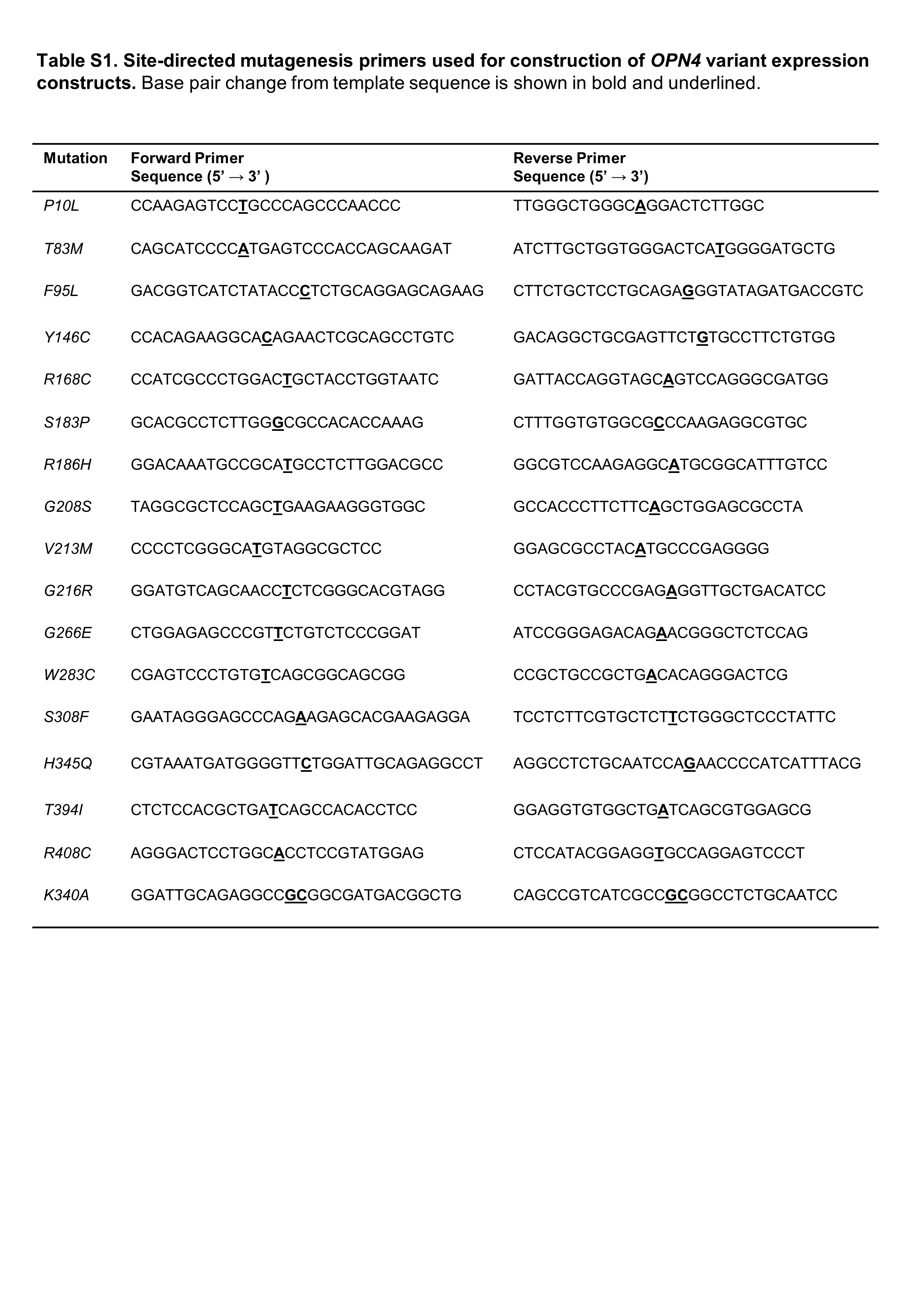

Supplement: Supplementary file 4 — Table S1. Site-directed mutagenesis primers used for construction of OPN4 variant expression constructs. Base pair change from template sequence is shown in bold and underlined. (TIF 866 kb) [file 18_2018_2813_MOESM4_ESM.tif]
